# 1731. Rezafungin Activity against *Candida* spp. and *Aspergillus* spp. Isolates Causing Invasive Infections Worldwide in 2021

**DOI:** 10.1093/ofid/ofac492.1361

**Published:** 2022-12-15

**Authors:** Cecilia G Carvalhaes, Paul Rhomberg, Paul Rhomberg, Greg Strand, Abby L Klauer, Mariana Castanheira

**Affiliations:** JMI Laboratories, North Liberty, Iowa; JMI Laboratories, North Liberty, Iowa; JMI Laboratories, North Liberty, Iowa; JMI Laboratories, North Liberty, Iowa; JMI Laboratories, North Liberty, Iowa; JMI Laboratories, North Liberty, Iowa

## Abstract

**Background:**

Rezafungin (RZF) is a once-weekly echinocandin (ECH) with a long half-life and front-loaded drug exposure. RZF is in development to treat candidemia and invasive candidiasis and prevent invasive fungal disease caused by *Candida*, *Aspergillus*, and *Pneumocystis* spp. We evaluated the *in vitro* activity of RZF, caspofungin (CSF), micafungin (MCF), and anidulafungin (ANF) against a worldwide collection of fungal isolates causing invasive infection.

**Figure:**
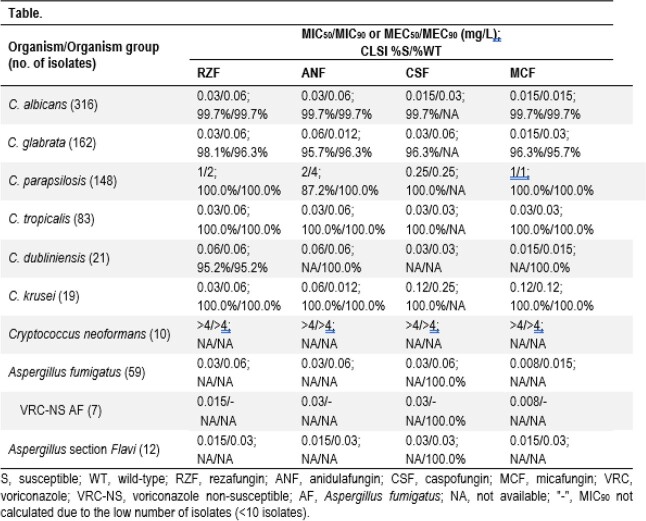

**Methods:**

830 isolates were collected from Europe (EU; 40.2%), North America (NA; 31.6%), Asia-Pacific (APAC; 15.3%), and Latin America (LA; 12.9%), identified by MALDI-TOF and/or sequencing, and tested by CLSI broth microdilution. Isolates included *C. albicans* (CA; 316 isolates), *C. glabrata* (CG; 162), *C. parapsilosis* (CP; 148), *C. tropicalis* (CT; 83), *C. dubliniensis* (CD; 21), *C. krusei* (CK; 19), *Cryptococcus neoformans* (CN; 10) *A. fumigatus* (AF; 59), and *A*. section *Flavi* (ASF; 12). CLSI criteria was applied, including the recently approved rezafungin provisional breakpoints against *Candida* spp.

**Results:**

RFZ inhibited 99.7% of CA, 98.1% of CG, 95.2% of CD, and all CP, CT, and CK (MIC_50/90_ in Table) at the susceptibility (S) breakpoint (BP). RZF had similar activity to the other ECHs against CA (99.7%S), CG (95.7-96.3%S), CT (100.0%S), CK (100.0%S), and CD (MIC_50/90_ range, 0.015-0.06/0.03-0.06 mg/L). Although CSF displayed lower MIC_50/90_ values (0.25/0.25 mg/L) than RZF (MIC_50/90_, 1/2 mg/L), MCF (MIC_50/90_, 1/1 mg/L), and ANF (MIC_50/90_, 2/4 mg/L) against CP, all ECHs but ANF (87.2%S) inhibited 100% of CP isolates at the respective BP. Only 1 CA (EU), 1 CD (EU), and 3 CG (NA) were non-S to RZF, while 1 CA (EU), 6 CG (5 NA, 1 APAC), and 19 CP (8 EU, 5 NA, 4 APAC, 2 LA) were ANF non-S. Limited activity was noted for all ECHs against CN (MIC_50_, > 4 mg/L). All AF isolates were inhibited by RZF at ≤ 0.06 mg/L, and ANF, MCF, and CSF at ≤ 0.12 mg/L. RZF (MIC range, 0.008-0.06 mg/L) and other ECHs (MIC range, 0.008-0.12 mg/L) were also active against 7 voriconazole non-S AF isolates (4 NA, 3 EU). RZF and other ECHs inhibited all ASF isolates at ≤ 0.06 mg/L.

**Conclusion:**

RZF was very active against *Candida* spp., AF, and ASF isolates causing invasive infections worldwide, including voriconazole non-S AF isolates and CP displaying non-S to ANF.

**Disclosures:**

**Cecilia G. Carvalhaes, MD, PhD**, AbbVie: Grant/Research Support|Cidara: Grant/Research Support|Melinta: Grant/Research Support|Pfizer: Grant/Research Support **Paul Rhomberg, BS, MT(ASCP)**, Cidara: Grant/Research Support|Pfizer: Grant/Research Support **Paul Rhomberg, BS, MT(ASCP)**, Cidara: Grant/Research Support|Pfizer: Grant/Research Support **Greg Strand, MLS (ASCP)CM**, Cidara: Grant/Research Support **Abby L. Klauer, BS**, Cidara: Grant/Research Support **Mariana Castanheira, PhD**, AbbVie: Grant/Research Support|Cidara: Grant/Research Support|GSK: Grant/Research Support|Melinta: Grant/Research Support|Pfizer: Grant/Research Support|Shionogi: Grant/Research Support.

